# The effect of protein mutations on drug binding suggests ensuing personalised drug selection

**DOI:** 10.1038/s41598-021-92785-w

**Published:** 2021-06-29

**Authors:** Shunzhou Wan, Deepak Kumar, Valentin Ilyin, Ussama Al Homsi, Gulab Sher, Alexander Knuth, Peter V. Coveney

**Affiliations:** 1 Department of Chemistry, Centre for Computational Science, University College London, London, WC1H 0AJ UK; 2 Computational Biology, Carnegie Mellon University in Qatar (CMU-Q), Doha, Qatar; 3 Hematology and Oncology Department, National Center for Cancer Care & Research, Hamad Medical Corporation, Doha, Qatar; 4 Interim Translational Research Institute, Hamad Medical Corporation, Doha, Qatar

**Keywords:** Nuclear receptors, Molecular medicine, Predictive markers, Genetics research, Breast cancer, Molecular dynamics

## Abstract

The advent of personalised medicine promises a deeper understanding of mechanisms and therefore therapies. However, the connection between genomic sequences and clinical treatments is often unclear. We studied 50 breast cancer patients belonging to a population-cohort in the state of Qatar. From Sanger sequencing, we identified several new deleterious mutations in the estrogen receptor 1 gene (ESR1). The effect of these mutations on drug treatment in the protein target encoded by ESR1, namely the estrogen receptor, was achieved via rapid and accurate protein–ligand binding affinity interaction studies which were performed for the selected drugs and the natural ligand estrogen. Four nonsynonymous mutations in the ligand-binding domain were subjected to molecular dynamics simulation using absolute and relative binding free energy methods, leading to the ranking of the efficacy of six selected drugs for patients with the mutations. Our study shows that a personalised clinical decision system can be created by integrating an individual patient’s genomic data at the molecular level within a computational pipeline which ranks the efficacy of binding of particular drugs to variant proteins.

## Introduction

Breast cancer is the most common cancer affecting women, and its mortality rate has increased significantly in the world during the past 25 years^1^. Given the prevalence of breast cancer, it is pertinent that we devise high-throughput experimental and computational methods that provide a comprehensive and holistic understanding of the cause of cancer. In the post-genomic “one size does *not* fit all” era, personalised medicine is surely the way forward, considering the improved ability provided by the methodology to inform treatments that would work effectively for individual patients. Advances in genomic profiling of breast cancer have led to the identification of several key mutations in the disease^2,3^. An in-depth understanding of the mechanisms of the disease requires not only a knowledge of the genome and its variants but the correct tools to fully interpret the knowledge. The pathways for the disease are routed via proteins, and it is their interactions that are amenable to treatment. This leads in turn to clinical decision support for personalised drug treatment. The lack of approved targeted treatments (other than mTOR inhibitors^4^ and anti-HER2 agents^5^), however, makes the genomic profiling of breast cancer less attractive compared with other tumours, such as lung cancer^6^.

An optimal selection of sequencing techniques is crucial to generate genomic libraries for specific patients, depending on sample size and the genomic targets. When interrogating a small region of DNA on a limited number of samples or genomic targets, Sanger sequencing is a good choice^7^. The estrogen receptor (ER) protein encoded by the ESR1 gene is expressed in about 70% of breast cancers^8^. ER also plays a vital role in classifying breast cancer subtypes and assigning therapeutic strategies; moreover, clinical research has established the central role of ER in the initiation and progression of breast cancers^9^. At least 62 ER mutations have been identified, of which most occur in the ligand-binding domain^8^. Several of the mutations are associated with ligand-independent ER activation or drug resistance. Experimental studies have revealed how some of the mutations affect the functions of ER, including changes of binding abilities for estradiol and drugs, abilities of dimerization, preferences of active and inactive states, and changes of interaction with cofactors and other proteins^8,10^. Computational studies also show that ERs can be constitutively activated in their apo form by some mutations^11^. Molecular Mechanics Poisson–Boltzmann Surface Area (MMPBSA) and Molecular Mechanics Generalized Born Surface Area (MMGBSA) approaches have also been used to study the binding free energy of ligands to the wild-type ER, although no overall correlation has hitherto been obtained between the calculations and the experimental results^12^.

The significance of sequencing and sequenced data lies in the identification of biomarkers and aberrations in the genome profiles of breast cancer patients. Identification of mutations in the ESR1 gene through genome profiling dates back to Weis et al. in 1996, who addressed the effect of mutations on the conformational dynamics of the ER receptor^13^, and to Zhang et al. in 1997, who identified three missense mutations in a cohort of 30 tumours^14^. In our study we identified genetic aberrations in 50 breast cancer patients from a population cohort in the state of Qatar using Sanger sequencing targeted on ESR1, and performed ESMACS (enhanced sampling of molecular dynamics with approximation of continuum solvent)^15,16^ and TIES (thermodynamic integration with enhanced sampling)^16,17^ binding free energy studies to understand the effects of these mutations in a manner that could be used in the development of novel therapeutic strategies to inhibit these ER mutants and substantially improve treatment outcomes^18^. We have extensively validated the ESMACS and TIES approaches by applying them to a variety of proteins with diverse sets of ligands. The studies show that these ensemble-based approaches can generate precise and reliable free energy predictions, while TIES method is also accurate^15–17,19–26^. We recently showed how such methods (i.e. ESMACS and TIES) can be used to assess functional and mechanistic impacts of mutations in the case of FGFR1 (fibroblast growth factor receptor 1) variants^27^. In the longer term, a related approach could be used to design new drugs which are resistant to such mutations.

## Materials and methods

Target gene sequencing for ESR1 gene was performed on the 50 tumour tissue samples collected from Qatari female patients with newly diagnosed estrogen receptor-positive breast cancer. The samples were preserved by formalin-fixed paraffin-embedded (FFPE) fixation. The study had been classified as “non-human subject research” by, and approvals granted from Institutional Review Board (IRB), Hospital Research Committee (HRC), Medical Research Center (MRC) and Hamad Medical Corporation (HMC) in Qatar. This ensured that we could deal with the anonymous tissue samples in accordance with relevant guidelines and regulations. Computational analysis was undertaken and missense SNP (single-nucleotide polymorphism) variants in the sequenced data were identified. The aim of the study was to identify possible ESR1 mutations within Qatari population, and to get an understanding of the drugs' response to the potential mutations for future drug development and clinical treatment. The study was not conducted for the purpose of treatment of the patients from whom the samples were collected. Among the identified variants, four significant missense SNPs were used in further analysis to understand their effect on protein-drug interactions and protein activation using computer based molecular dynamics simulations. Because of the prospective nature of the modelling study, all of the four important SNPs are investigated, even though some of them have unclear chromatograms (see Table S3 in the Supplemental Material) and their statistical significance needs to be evaluated with large datasets.

### Genome sequencing

Fifty breast cancer samples were collected from a population cohort of breast cancer patients in the state of Qatar at Hamad Medical Corporation (HMC) and were subjected to Sanger sequencing. The Sanger sequencing method was applied to the ten coding exons of the ESR1 gene in these samples to detect aberrant mutants. For sequencing, genomic DNA was isolated from formalin-fixed, paraffin-embedded tissue by Maxwell 16 FFPE Tissue LEV DNA Purification Kit (Promega). The quality and quantity of the DNA was checked by NanoDrop 2000c Spectrophotometer (Thermo Scientific) and agarose gel electrophoresis. Specific primers for the coding exons of the ESR1 gene (Transcript ID: ENST00000440973.5) were designed by Primer3web software, v4.0.0. The coding exons were amplified by PCR using Maxima Hot Start PCR Master Mix (Thermo Scientific) and purified by Gene JET PCR Purification kit (Thermo Scientific). Cycle sequencing was carried out using the BigDye Terminator v3.1 cycle sequencing kit. Sequencing reaction products were purified by the BigDye XTerminator purification Kit and analysed on an ABI 3500 Genetic Analyzer (Applied Biosystems). All procedures were carried out according to the manufacturers’ instructions. Finally, sequenced data was generated in AB1 format files.

Thereafter, variant-calling computational analysis was performed on the sequenced data and missense variants were identified. These missense SNP variants in the sequenced data were called by SeqScape Software 3 (applied biosystems). A mutation report was generated for each patient. Chromatogram analysis was performed on the sequenced data to detect artefacts such as mis-called-nucleotides and aberrations. A list of SNPs (synonymous and nonsynonymous) was thus generated consisting of the patient number, mutation, and its novelty or known status based on variant databases: dbSNP^28^, Ensemble^29^, TCGA (https://www.cancer.gov/tcga), gnomAD^30^, MOBCdb^31^, 1000 Genomes^32^, TOPMed^33^, ExAC^34^, COSMIC^35^, HGMD^36^ and ESP (https://esp.gs.washington.edu/drupal/). All new nsSNPs were analysed using StSNP^37^.

### Molecular dynamics based investigation of protein-drug interactions

Mutations obtained from sequencing analysis were subjected to a modelling and simulation study in order to understand the effect of these variants on the binding affinity of drugs to ER. To validate our computational approaches within the current molecular systems, a control study was also conducted, in which simulations were performed for three mutations—L387A, Y537S and D538G—of which experimental binding affinities were available^10,38^.

We used ensemble-based ESMACS and TIES for the free energy calculations. Extensive studies have confirmed that the most effective and reliable computational route to reproducible predictions using MD simulation can be achieved using ensemble methods^23,39–41^. A set of independent MD simulations are employed to obtain the required averages and associated uncertainties. The protocols of 25 replicas for ESMACS and 5 replicas for TIES, with 4 ns production runs, were established in our previous studies^15,17,20,23,39,42,43^, in which the number of replicas and the duration of the production runs were varied, and the results were compared between the ensemble runs and the “long time duration” single trajectory simulations. Our work demonstrates compellingly that the ensemble approach produced more precise and reproducible predictions than long simulations, even though the latter were several times longer in temporal duration than the entire ensemble simulation. The variations of the results from ensemble simulations are typically larger than those from single long simulations^15,20,23,39^, indicating better conformational sampling achieved from the former. The following simulation study methodology was executed.

#### Molecular models

**Figure 1:**
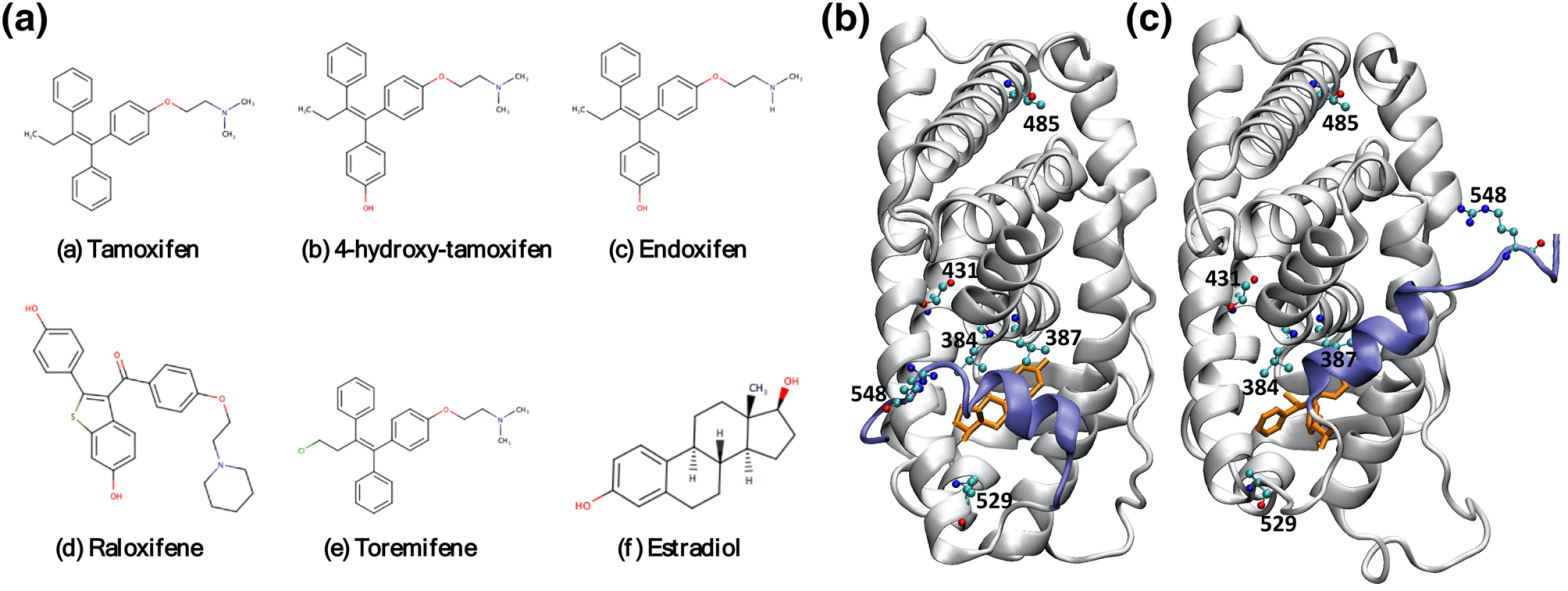
Chemical structures of the 6 ligands that have been investigated (**a**), and positions of the mutations identified from 50 breast cancer patients of Qatari nationals, in both the active (**b**) and inactive (**c**) conformations. The PDB code of the active conformation crystal structure is 1QKU, and the inactive conformation is 3ERT. The ligands presented in the crystal structures are represented as stick in orange, the protein is shown as cartoon in silver. The helix 12 (H12) is highlighted in blue, which shows different orientations in the active (**b**) and inactive (**c**) conformations.

Binding affinities were obtained for 5 ER drugs or drug metabolites: toremifene (TOR), endoxifen (EDO), raloxifene (RAL), 4-hydroxy-tamoxifen (4-OHT) and tamoxifen (TMX), and the natural ligand estrogen (E2) for ER (Fig. 1a). The ligand-binding domain of the estrogen receptor is an α-helical bundle, of which several helices, particularly helix 12 (H12, see Fig. 1b,c), are known to be crucial for activity. At the active conformation, the H12 helix caps the ligand binding cavity (Fig. 1b) and its position is a prerequisite for coactivator recruitment to the activation function 2 (AF-2) cleft. In the inactive conformation, the H12 helix occupies the AF-2 cleft (Fig. 1c) preventing the coactivator to interact with the ER and to trigger transcription activity.

Two x-ray structures of the estrogen receptor, PDB codes 1QKU^44^ and 3ERT^45^, were used for this study, which represent the active and inactive forms of the protein, with the H12 helix at different positions (Fig. 1b,c). The ER structure of the former PDB model is complexed to 4-OHT, whereas the latter is bound to the native E2. 4-OHT and E2 bind to ER in different conformations. 4-OHT, an antagonist, displaces the usual position of the H12 helix so that the ER is found in an inactive conformation. E2, as the natural ligand for ER, fits in the binding pocket without sterically hindering the H12 helix, and thus the E2-ER complex exists in an active conformation (Fig. 1b). The complex structures for TOR, EDO and TMX were generated by replacing the 4-OHT inhibitor in 3ERT, after overlapping the common scaffold of the ligands. The coordinates of Ral in PDB 2QXS^46^ were used to build the model of RAL after aligning the two PDB structures 2QXS and 3ERT. All corresponding crystallographic water molecules in 1QKU and 3ERT were retained.

#### ESMACS studies

Enhanced sampling of molecular dynamics with approximation of continuum solvent (ESMACS)^15,16^ studies employed an ensemble molecular dynamics approach which consists of 25 replica simulations. For each replica, the same initial coordinates were used for a given ligand-receptor complex, with different initial velocities randomly assigned to the atoms according to a Maxwell–Boltzmann distribution at 50 K. The systems were first heated over a period of 60 ps to 300 K, followed by 2 ns equilibration and 4 ns production runs for each replica. All simulations are performed in an isothermal-isobaric (constant temperature and constant pressure) ensemble using periodic boundary conditions. Free energy was evaluated approximately on the basis of the MMPBSA (molecular mechanics Poisson–Boltzmann surface area) method applied on a set of conformations from ensemble molecular dynamics simulations (see more details in the Supplemental Material).

#### TIES-PM studies

We have recently extended our TIES (thermodynamic integration with enhanced sampling) approach^17,23^ to study the free energy changes caused by protein mutations, a TIES variant we call TIES-PM^19^. We have established a standard protocol for TIES-PM, in which thirteen windows, consisting of the two endpoints representing the two physical states (WT and mutant ERs) and 11 intermediate states, are simulated for the alchemical process of protein mutation. The intermediate windows are mixtures of the two physical states that consist of the appearing and disappearing parts of the residues (see Supplemental Material for more details). Simulations were performed for both ligand–protein complexes and apo-proteins. Five replicas were used for each window, from which the energy deviations and the statistical errors were calculated^19,27^. The binding free energy differences were then calculated as the difference of the alchemical free energy changes in the apo-proteins and ligand bound complexes.

TIES-PM calculations involve an alchemical mutation between two amino acids. Four residue mutations identified in the current sequencing study were selected for the TIES-PM study: L384V, L387R, K529N and R548P. Although ESMACS and TIES (including TIES-PM) have been adequately validated for a variety number of protein systems, a control study is preferable here as no experimental data is available to support our predictions. We perform TIES-PM and ESMACS simulations for L387A, Y537S and D538G as an internal control. It should be noted that while L387A occurs inside the binding pocket (“local”), the other two mutations occur away from the binding pocket (“remote”). Our previous study has shown that alchemical methods, even with enhanced sampling approaches, may not be able to predict the binding free energy changes for such remote mutations^19^. Some of these mutations involve perturbing the net charge of the system, which requires additional calculations to take into account the resulting finite size electrostatic corrections to the free energy^20,47^.

#### Simulations

The binding affinity calculator (BAC)^48^ software tool was used to perform ESMACS and TIES studies. BAC constitutes a computational pipeline built from preparation and setup of the simulations, including parametrization of the compounds, solvation of the complexes, electrostatic neutralization of the systems by adding counterions and generation of configurations files for the simulations. The Amber package^49^ was invoked for the setup of the systems and analyses of the results, and the MD package NAMD2.12^50^ was used throughout the equilibration and production runs of all simulations. The AMBER ff99SBildn force field^51^ was used for the protein, and TIP3P was used for water molecules. Parameters for the ligands were produced using the general AMBER force field (GAFF)^52^ with Gaussian^53^ calculations at the Hartree–Fock level with 6-31G** basis functions. The restrained electrostatic potential (RESP) module in the AMBER package^49^ was used to calculate the partial atomic charges for the ligands. All of the ligands are electrostatically neutral except Ral which has a +1e net charge. All systems were solvated in orthorhombic water boxes with a minimum distance of 14 Å between box boundary and the ligand–protein complex. Standard protocols for ESMACS^15^ and TIES^17^ have been applied, in which simulations of multiple replicas were performed with identical initial conditions other than their initial velocities, which were drawn randomly from a Maxwell–Boltzmann distribution. Energy minimisation and 2 ns equilibration were conducted before 4 ns production runs were performed for each replica of the ESMACS and TIES-PM studies. Trajectories were recorded every 10 ps during the production runs for further analyses.

All simulations were run on the BlueWaters supercomputer at the National Center for Supercomputing Applications of the University of Illinois at Urbana–Champaign (https://bluewaters.ncsa.illinois.edu). Simulations of all replicas in an ensemble were executed concurrently, and completed in essentially the same amount of wall-clock time as that for one replica. For one single replica, a 2 ns equilibration and 4 ns production MD simulation took 15.7 h on 2 nodes (64 cores) of BlueWaters.

## Results and discussion

### Sequencing analysis

From our sequencing study, 22 mutations (Supplemental Material) were identified, of which six were nonsynonymous and in the ligand-binding domain, as shown in Table 1 and Fig. 1b,c. 7 of them were silent mutations, of which some were observed at relatively high frequencies (Table S2). Among these 22 mutations, 14 mutations were identified to be novel with no annotations available in nucleotide variants repositories. On the other hand, 8 mutations were found to be known with their respective annotations accessible in variant databases such as dbSNP. Corresponding frequencies of mutations in the studied 50 breast cancer samples were computed to understand their occurrence and cluster pattern across the analysed patient cohort (see the Supplemental Material). Such studies of detecting mutation occurrence patterns could be used for advance statistical analysis, where the identified mutations’ presence is not only studied in patient samples from a specific region but, also for its uniqueness and ubiquity in other assessed population cohorts. Furthermore, identification of unique and prevalent mutations in diverse ethnic populations will play a vital role toward the goal of precision medicine in pharmacology^54^. Along these lines, data curation and mining were performed on breast cancer data available in public repositories to validate the novelty of the mutations identified with clear chromatograms (Table S3; Supplemental Material) in the Qatari breast cancer patient cohort studied. Among the curated databases, Ensemble is a comprehensive collection of variant information from multiple sources such as dbSNP, COSMIC, ESP and HGMD-PUBLIC. Moreover, the Ensemble database also provides evidences of the mutations’ significance and validity from large-scale sequencing catalogues of human mutations and genotype data such as 1000 Genomes project, ExAC, TOPMed and gnomAD. From the analyses executed, it was deduced that the identified novel mutations hold their uniquity among the cancer data present in the databases. A total of 226 synonymous and 373 non-synonymous mutations in ESR1 gene protein coding region were observed in the Ensemble repository, and their respective evidences were validated from 1000 Genomes project, ExAC, TOPMed and gnomAD. None of our identified novel mutations were reported in the analysed databases. Furthermore, novel mutations identified in Qatar cohort were not observed in the TCGA and MOBCdb multi-omics breast cancer databases. A total of 17 samples with non-synonymous and 12 samples with synonymous somatic mutations in ESR1 gene were discerned in TCGA database—again, none of the novel mutations were observed in the studied TCGA samples. Additionally, ClinVar^55^ database was mined to check the presence and clinical significance (if any) of the identified novel mutations; 13 mutations belonging to ESR1 gene with likely-pathogenic and pathogenic clinical significance status were noticed—no novel mutations from the analysed Qatar cohort were detected. Further, from the comprehensive Open Targets Consortium^56^ consisting of evidence from genetics, genomics, transcriptomics and target-disease associations, we noticed that the identified novel mutations were not present in the Consortium database. Thus, from the available public data it can be concluded that the novel mutations identified in the Qatar breast cancer patient cohort have not been studied previously with any reported clinical significance. It should be noted that some mutational signatures are annotated as possible artefacts in Table 1 because of the chromatogram quality from the FFPE samples.

**Table 1:** SNP missense variants obtained from Sanger sequencing study.

Residue number	Reference/mutant	Status	Mutant characterization	Comments^a^
384	L/V	Novel	real	Binding pocket
387	L/R	Novel	artefact	Binding pocket
431	T/A	Known	artefact	No direct interaction with the ligand
485	T/I	Novel	real	Far from binding site; may be important for domain-domain interaction or dimerization
529	K/N	Novel	artefact	At the C-terminal of helix H11, which links to the N-terminal of helix H12; may be important for the orientation of H12; not very far from the ligand (~ 7 Å)
548	R/P	Novel	artefact	At the C-terminal end of helix H12; may be important for the orientation of H12

^a^The assumptions of their effects are based solely on the positions of the mutations in the static crystallographic structures (Fig. 1b,c). More studies on structures, dynamics and energetics will be required to confirm or refute these assumptions.

Two of the 22 mutations are at the binding site—L384V and L387R—while two located at or near helix 11 or 12—K529N and R548P—are important for the orientation of helix H12 (Fig. 1b,c). Interestingly, no mutations were found between amino acids 534–538, a region where most mutant residues are reported to cluster^18^.

These four mutations, L384V, L387R, K529N and R548P, are directly involved in ligand binding or protein activation, and were further investigated by our ESMACS and TIES approaches. The other two mutations, T431A and T485I, occur away from the ligand binding site or the helices H11/H12; these are not expected to affect the ligand binding or protein activation directly. They may modulate the protein stability and/or protein–protein interactions via induced allosteric conformational changes occurring over a wide range of space and time scales. The spatial and temporal scales are greater than standard atomistic molecular dynamics simulations can access^19^, and hence no further investigation was performed for these mutations using molecular dynamics modelling approaches.

### Molecular dynamics study result

In the control study, the binding free energies of E2 were calculated for three mutations—L387A, Y537S and D538G, and compared with the experimental data^10,38^ (Table 2). The same binding assay was performed in the two publications^10,38^, with different mutations. They both measured the dissociation constant of E2 with the wild-type ER, with results differing by more than 2 folds (equivalent to ~ 0.5 kcal/mol difference in the binding free energy). It highlighted the uncertainties of experimental measurement, and contributed to the differences between the calculations and experiments. For the local mutation L387A, the calculated binding free energy differences from ESMACS and TIES agree directionally with that from the experimental data; that is, both calculations and experiment show that the mutation weakens the binding of E2 to the protein. For the two remote mutations Y537S and D538G, the ESMACS approach correctly predicts the weakened binding. TIES approach, however, cannot predict such changes in binding affinities. As reasoned in our previous publication^19^, the effect of remote mutants affects the binding of a compound indirectly through an allosteric mechanism. TIES only samples local conformational changes which are not affected by remote mutations^19^. Both ESMACS and TIES work well for local mutations; we therefore focus the free energy predictions on two mutations: L384V and L387R. It should be noted that while TIES approach is theoretically accurate, ESMACS invokes a few approximations in the energy estimations. ESMACS can generate rankings reasonably well for a set of compounds based on their binding affinities, but the differences of the affinities between pairs of compounds are not accurate.

**Table 2:** Relative binding free energies $\Delta \Delta G~ = ~\Delta G_{{binding}}^{{mut}}  - \Delta G_{{binding}}^{{WT}}$ for six ligands with two ER mutations—L384V and L387R—from ESMACS and TIES approaches.

Ligand	∆∆G_ESMACS_		∆∆G_TIES_		pdb
	L384V	L387R	L384V	L387R	
4-OHT	1.3 ± 1.1	1.0 ± 1.3	2.2 ± 0.4	5.1 ± 0.5	3ert
EDO	0.3 ± 1.1	0.9 ± 1.3	2.0 ± 0.4	4. 8 ± 0.5	3ert
RAL	3.9 ± 1.9	3.6 ± 2.0	2.2 ± 0.4	6.1 ± 0.9	3ert
TMX	0.6 ± 1.0	3.4 ± 1.2	2.2 ± 0.4	4.7 ± 0.6	3ert
TOR	0.2 ± 1.0	4.3 ± 1.3	2.2 ± 0.5	4.6 ± 0.6	3ert
E2	1.7 ± 1.2	1.3 ± 1.3	2.2 ± 0.3	5.2 ± 1.8	1qku

Control E2	L387A	Y537S	D538G	pdb	
∆∆G_ESMACS_	3.9 ± 1.3	1.0 ± 0.9	1.0 ± 0.9	1qku	
∆∆G_TIES_	1.8 ± 0.2	− 0.4 ± 0.5	− 0.5 ± 0.6	1qku	
∆∆G_exp_	0.5 ± 0.2^10^	1.1 ± 0.4^38^	1.2 ± 0.4^38^	–	

The calculations for the three mutations taken from the literature—L387A, Y537S and D538G—are presented as a control. The Poisson-Boltzmann (PB) free energy methods were used in the predictions of the ESMACS free energies, while alchemical approach was used for TIES. All energy values are in kcal/mol.

#### Free energy calculations with ESMACS for mutations identified in Qatari population

The predicted binding affinities of L384V and L387R from ESMACS were compared with the wild-type results (Table 2). Other mutations (Table 1) were not included as they are positioned away from the binding pocket and do not have any direct interaction with the ligands. Some mutations, such as K529N and R548P, are located at a key position for the orientation of the helix H12 (Fig. 1b,c), and are expected to play a role in the active-inactive conformational changes. Their potential roles are investigated by the TIES approach (see the next section).

L384V and L387R induce resistance in all of the studied ligands, evidenced by the positive relative binding free energies for the mutated ERs compared with the wild-type ER with the corresponding ligands. L384V and L387R occur in the binding pocket and directly interact with the ligands. The L387R mutation, in particular, introduces not only steric bulk but a net electrostatic charge change. It induces significantly larger free energy changes for the ligands TMX and TOR than the mutation L384V does (Table 2). Large changes in the size of the residues and the charge distributions can confer resistance to and even completely block access to the ligands. To the best of our knowledge, there are no experimental data reported for the changes in binding affinity induced by these specific mutations. Other mutations, however, have been reported to weaken the binding of estradiol when they occur at the binding site^10^, including a mutation (L387A) occurring at the same position as L387R studied here.

#### Free energy calculations with TIES-PM for mutations identified in Qatari population

The binding affinities of two mutations L384V and L387R were investigated by the TIES-PM approach (Table 2). The L387R mutation involves a net charge change, and hence a finite-size effect needs to be taken into account^20,47^.

Both mutations induce resistance, which is in line with the ESMACS predictions. The L384V mutation weakens the binding for all of the ligands by 2.0–2.2 kcal/mol universally. The L387R mutation has a higher impact on the ligand binding, reducing the binding affinities by 4.6–6.1 kcal/mol. In drug discovery, a rule of thumb to consider compounds for further development is to select those with dissociation constants (*K*_*d*_) in the millimolar to micromolar range, usually with an equivalent binding affinity more negative than − 6.5 kcal/mol^57^. The large changes for the L387R mutation make the binding free energies all around or less negative than − 6 kcal/mol for the ligands investigated here. This means that L387R mutation is likely to block the binding of all these ligands, including the native estradiol.

#### Conformation free energy changes with TIES-PM for mutations identified in Qatari population

**Figure 2:**
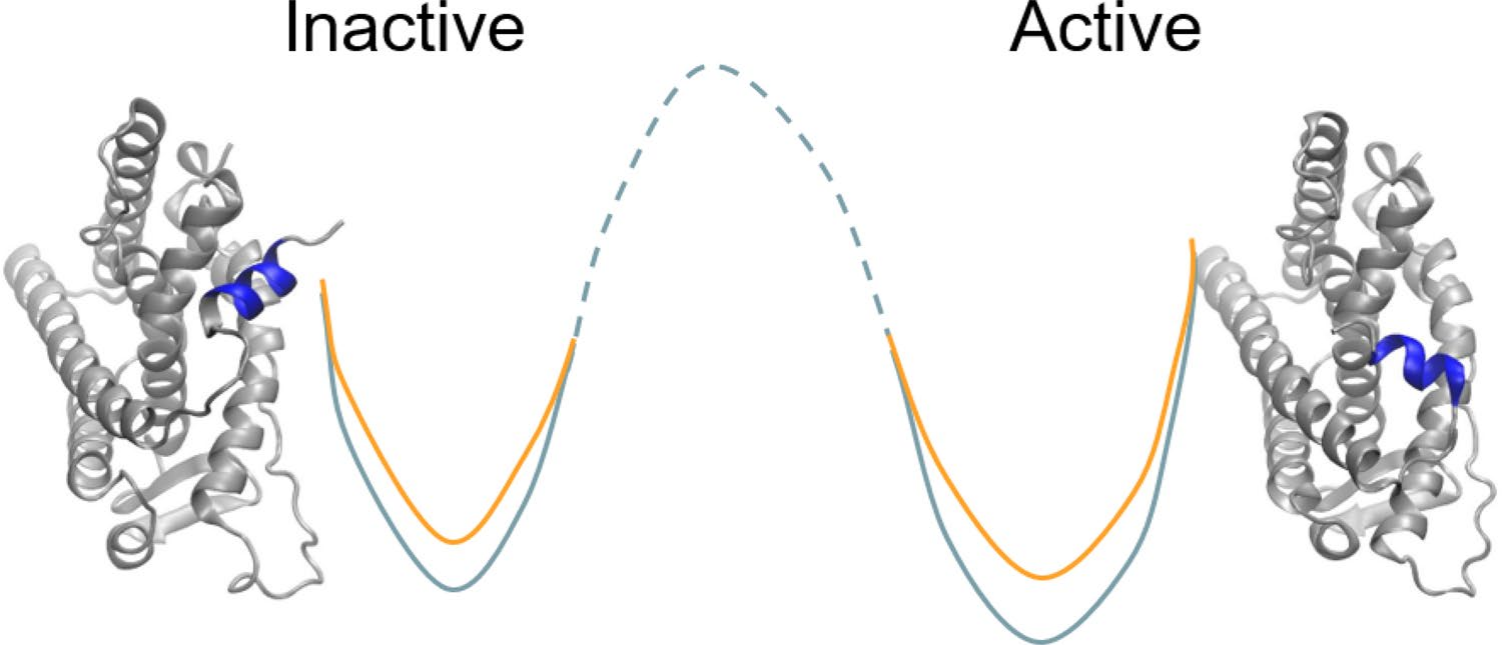
Conformational free energy changes of the active and inactive states due to mutation. The mutations change the relative energy differences between the two states, and hence shift the balance between them. The 2D energy surface illustrates an example of the energy changes at the two states, from wild-type (orange) to mutant (light blue), rendering the active state more favourable for the mutant protein than the wild-type. It should be noted that the 2D energy surface is an illustration as the free energy difference between the active and inactive conformations of the wild type, and the energy barrier between them (the dashed line) are unknown.

The relative conformational free energy changes were investigated by the TIES-PM approach (Fig. S1b and Eq. S5 in Supplemental Material). Previous studies have shown that mutations can result in a change of activity for protein kinases. A gatekeeper mutation in fibroblast growth factor receptors (FGFRs), for example, has been shown to enhance the kinase activity using the later named TIES-PM approach^27^. The estrogen receptor exists in at least two conformational states: active and inactive (Figs. 1b,c and 2). The receptor is likely to favour the inactive conformation at the physiological condition. Mutations may change the intrinsic equilibrium between the active and inactive states without ligand binding. Studies of large conformational changes are usually beyond the scope of standard all-atom molecular dynamics simulation^19^; while “accelerated” MD simulations can provide a free energy profile between the two states^27^, they come with large uncertainties owing to the nature of the approximations used.

The TIES-PM approach can deliver accurate and precise predictions, and is used here to investigate the relative binding free energy changes in the two states caused by protein mutations (Fig. 2). The apo forms of the protein are simulated at both the active and inactive states. For each state, TIES-PM is performed to alchemically transfer the protein from wild-type to mutant form (Fig. S1b). The alchemical free energy changes are then used to calculate the conformational free energy difference ∆∆G (Eq. S5). ∆∆G is a physical property which is used here to quantify the changes of the preference for the two states. The calculations show that the L384V and K529N mutations confer a moderate change on the relative stability of these two protein states, rendering the active state slightly more favourable, or less unfavourable, for the mutant protein than the wild-type (Table 3). By contrast, the L387R and R548P mutations have a large impact on the preference of the two states, making the active state significantly more favourable, or less unfavourable, for the mutant proteins than for the wild-type. It should be noted that the finite size electrostatic corrections contribute importantly to these calculations and improve the predicted free energy changes significantly.

**Table 3:** Relative conformational free energy changes $\Delta \Delta G = \Delta G_{{TIES}}^{{act}}  - \Delta G_{{TIES}}^{{inact}}$ between the active and inactive states upon a mutation.

Mutation	Active		Inactive		ΔΔG
	ΔG_TIES_	ΔG_FS_*	ΔG_TIES_	ΔG_FS_*	
L384V	2.7 ± 0.3	–	1.1 ± 0.4	–	1.6 ± 0.4
L387R	− 29.6 ± 0.4	54.6	− 37.8 ± 0.4	57.0	5.8 ± 0.5
K529N	30.8 ± 0.2	− 52.5	32.4 ± 0.7	− 55.3	1.2 ± 0.7
R548P	60.0 ± 0.7	− 52.5	56.3 ± 0.5	− 56.9	8.2 ± 0.8

ΔΔG > 0 means that the free energy change in the active state is larger than that in the inactive state (Fig. 2). All energy values are in kcal/mol.

*Finite size correction, related to the size of simulation box; the error associated is negligible.

#### Structural base for the preference of active state

**Figure 3:**
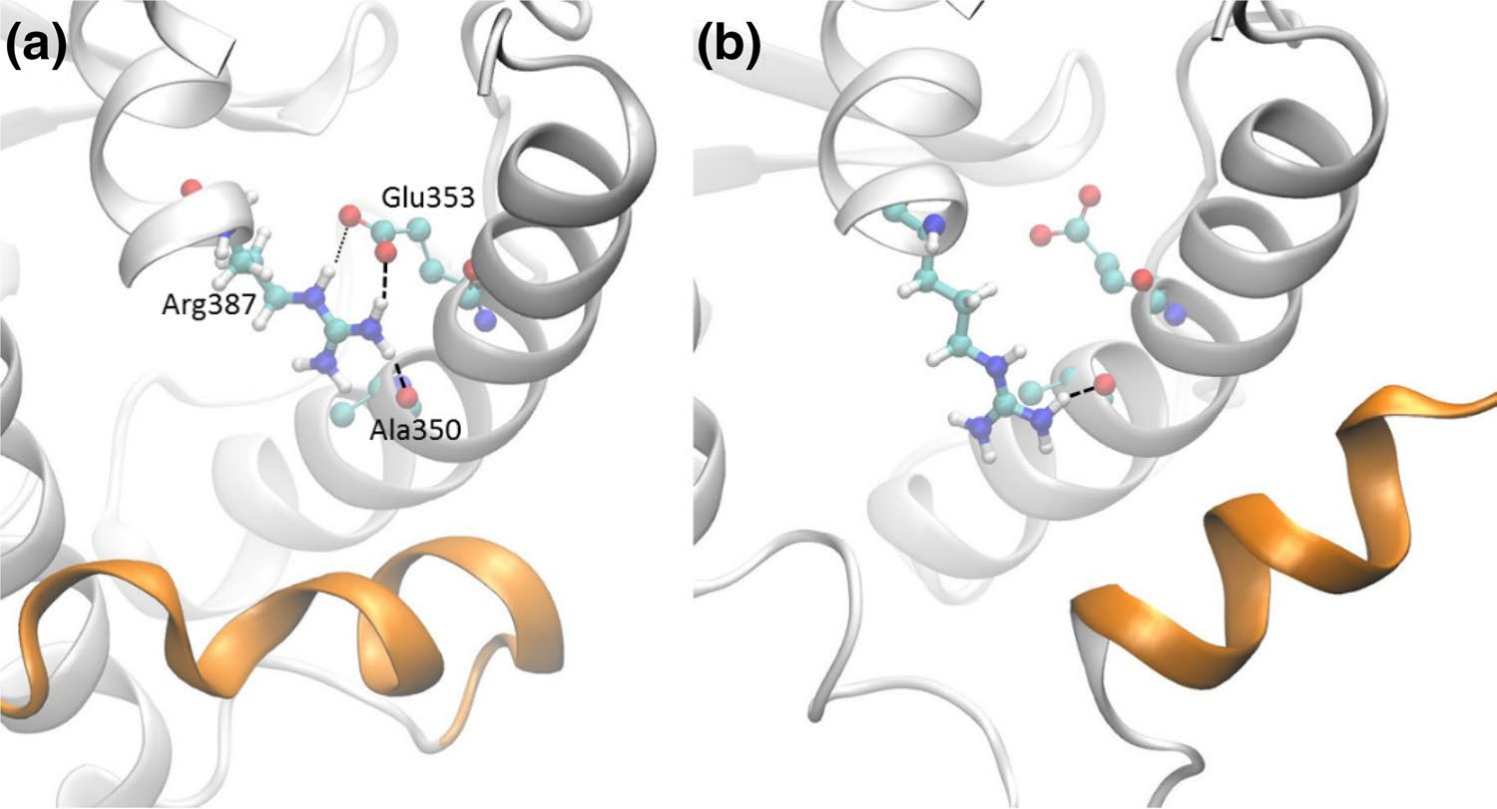
Formation of hydrogen bonds between the mutant residue 387 with other residues within the helix 3 (H3) at the active (**a**) and inactive (**b**) states. At the active state, Arg387 forms one stable hydrogen bond (bold dashed lines) with residues Ala350 and Glu353 each, and an additional one (light dotted line) with Glu353, which appears in ~ 65% of the entire simulations. At the inactive state, the side chain of Arg387 only forms one stable hydrogen bond with Ala350 (side chain of Glu353 forms hydrogen bonds with Arg394 instead, with 126% frequency of occurrence; the frequency is 15% in the active state). The helices H4, H8 and H9 are removed from both figures for reasons of clarity.

Our free energy results showed that, thermodynamically, all of the 4 mutations prefer the active state over the wild type. For the wild-type protein, the residue Leu387 participates in hydrogen bonding (see more details in the Supplemental Material) only via its main chain atoms to form the α-helix structure. It enjoys a similar pattern of hydrogen bonding, with 79% and 69% frequencies of occurrence in the active and inactive states, respectively. The substitution of Leu387 with a positively charged, polar residue Arg387 creates more hydrogen bonds via its side chain atoms (Fig. 3). In the active state, the side chain atoms form hydrogen bonds with residues in helix 3, with a frequency of 222% (2.22 hydrogen bonds on average, see Fig. 3a). In the inactive state, the H12 helix packs with helices H3 and H5, and slightly changes the orientation and conformation of the latter. The residue Glu358 on H3, which maintains stable hydrogen bond with Arg387 in the active state, forms hydrogen bond with Arg394 instead in the inactive conformation. As a result, the side chain of Arg387 only maintain one hydrogen bond, with a frequency of 103%, with residues in helix 3 (see Fig. 3b). The more stabilising hydrogen bonds in the active state shift the balance between the active and inactive forms, making the L387R variant thermodynamically preferable to the active state.

For the other mutations, the reasons for the free energy changes are more subtle, and cannot be ascribed to any single, dominant contribution. It is likely, however, that for the L384V variant, a less bulky substituent reduces the steric hindrance of the H12 helix when the protein is in the active conformation (Fig. 1b). For the mutations K529N and R548P, which are both located at the surface of the protein and involve net charge changes, it is likely that the stability of the protein is affected mainly by electrostatic interactions and solvation effects. The stability of the protein is ﻿probably attribute to the conformations and energetically of the side chains, as no significant changes are observed in the residue-wise root mean square fluctuations for the main chain atoms. For these mutations, there may not be one single indicator that explains why either the active or inactive state is favoured.

## Conclusions

Our study, performed on 50 breast cancer patients in a Qatari population cohort, furnishes a holistic understanding of the effect of deleterious mutations on the effectiveness of prevalent breast cancer drugs available today. Moreover, although the present study is based on a small set of 50 breast cancer patients, it demonstrates the power of patient-specific medical approaches in treating breast cancer as it reveals the presence of uncommon mutations among patients within one local and small geographical region. The sequencing study identified several mutations among breast cancer patients in Qatar. Some of these mutations are of considerable interest, and have not been previously reported in the public repositories of cancer data. In the future, in tandem with the validation of the identified novel mutations in the Qatari population cohort from publicly available consortiums, we would like to collect more samples, both within Qatar and worldwide, to perform computational analysis and determine whether these novel mutations are specific to the Qatari population and to investigate their more general importance.

Based on this genomic analysis, we then performed a rigorous and in depth molecular modelling study of the estrogen receptor with sequential variations obtained from the gene sequencing study in this project. The molecular modelling approaches were applied to the newly identified mutations in the ligand-binding domain of the receptor. The predicted binding free energies provide a clear explanation for the effects of these mutations. The mutations at the binding site, L384V and L387R, induce resistance to the drugs studied here; the mutations L387R and R548P play an important role in the activation of the estrogen receptor. This methodology may in the future be employed as the basis for a clinical decision support tool for patient specific drug treatment: the combination of rapid genome sequencing and binding affinity calculations offers a powerful and reliable way to provide patient specific treatment regimens. Along similar lines, these approaches may also be used to design new drugs which inhibit the development of resistance in the target proteins.

## Supplementary Information

Supplementary Information 1.

Supplementary Information 2.

Supplementary Information 3.
