# Supplementary material for: The effect of protein mutations on drug binding suggests ensuing personalised drug selection
Source: Sci Rep. 2021 Jun 29;11:13452. doi: 10.1038/s41598-021-92785-w (PMC8241852; doi:10.1038/s41598-021-92785-w)
Supplement: Supplementary file 2 — Supplementary Information 2. [file 41598_2021_92785_MOESM2_ESM.pdf]

Supplemental Material for

The Effect of Protein Mutations on Drug Binding Suggests Ensuing Personalised Drug Selection

Shunzhou Wan[a‡], Deepak Kumar[b‡], Valentin Ilyin[b], Ussama Al Homsi[c], Gulab Sher[d], Karl Richard Alexander Knuth[c] and Peter V. Coveney[a]*

[a] Centre for Computational Science, Department of Chemistry, University College London, London WC1H 0AJ, United Kingdom.

[b] Computational Biology, Carnegie Mellon University in Qatar (CMU-Q), Doha, Qatar.

[c] Hematology and Oncology Department, National Center for Cancer Care & Research, Hamad Medical Corporation, Doha, Qatar.

[d] Interim Translational Research Institute, Hamad Medical Corporation, Doha, Qatar.

[‡] S.W. and D.K. contributed equally to this work.

[*] Corresponding author: p.v.coveney@ucl.ac.uk

The main article describes the identification of deleterious mutations in the estrogen receptor 1 gene (ESR1) from a cohort of 50 breast cancer patients in the state of Qatar, and the prediction of protein-drug interactions using computer-based molecular dynamics simulation. We used two approaches to investigate the protein-drug interactions, namely ESMACS (enhanced sampling of molecular dynamics with approximation of continuum solvent) and TIES (thermodynamic integration with enhanced sampling). The Supplemental Material describes the free energy methods in more detail, together with all of the mutations identified in the ESR1 gene.

Free Energy Methods

Given the rapidly growing popularity of free energy calculations in drug development and personalized medicine, it is all the more necessary to ensure that the predictions are reproducible[1]. The detailed description of the ESMACS and TIES methods has been provided in recent publications[2,3]. For the reader's convenience, we briefly summarise these methods here.

Thermodynamic cycle. The free energy calculation is carried out in the context of a thermodynamic cycle as depicted in Figure S1. For binding free energy differences (Figure S1a), the horizontal legs are the binding process of a ligand to the same protein with slightly different sequencing, here being the wild-type and mutant ER. The free energy of ligand (lig) binding with protein (pro) in solvent can be evaluated as:

$$\Delta G_{binding} = G_{com} - G_{pro} - G_{lig} \qquad Eq.\ S1$$

where G_i is the free energy of component *i* which corresponds to either complex (com), ligand (lig), or protein (pro). ESMACS approach is based on this equation, in which G_i is calculated from a set of structures from MD simulations.

The vertical legs in Figure S1a are alchemical processes during which a molecule is transferred from one to another. Here the alchemical approach is used to calculate the binding free energy differences of a given ligand to the wild-type and mutant ERs, and the binding free energy difference is calculated from

$$\Delta\Delta G^{binding} = \Delta G_2^{binding} - \Delta G_1^{binding} = \Delta G_{com}^{alch} - \Delta G_{pro}^{alch} \qquad \textit{Eq. S2}$$

Consider two systems, A and B, with potential energies U_A and U_B. To apply thermodynamic integration, a series of intermediate states, with potential energies $U(\lambda)$, are introduced, which links the two physics states (the two systems), A and B. λ is a coupling parameter such that λ=0 and λ=1 correspond to the initial and final thermodynamic states A and B, respectively. The potential energy of an intermediate state λ is taken as a combination of the initial and final potential energies as follows:

$$U(\lambda) = (1-\lambda)U_A + \lambda U_B \qquad \textit{Eq. S3}$$

The free energy ΔG_i^{alch} $(i = pro/com)$ of the alchemical process is given in thermodynamic integration (TI) as:

$$\Delta G^{alch} = \int_0^1 \langle \frac{\partial U(\lambda)}{\partial \lambda} \rangle_\lambda \, d\lambda \qquad \textit{Eq. S4}$$

Here $\langle\cdots\rangle_\lambda$ denotes an ensemble average over configurations representative of an intermediate state λ.

Similarly, wild-type and mutant ER have different free energy changes, ΔG_{WT}^{conf} and ΔG_{WT}^{conf}, when conformations are changed from active to inactive. The relative changes upon mutations can be calculated with a thermodynamic cycle (Figure 1b) as:

$$\Delta\Delta \mathrm{G} = \Delta G_{mut}^{conf} - \Delta G_{WT}^{conf} = \Delta G_{TIES}^{inact} - \Delta G_{TIES}^{act} \qquad \textit{Eq. S5}$$

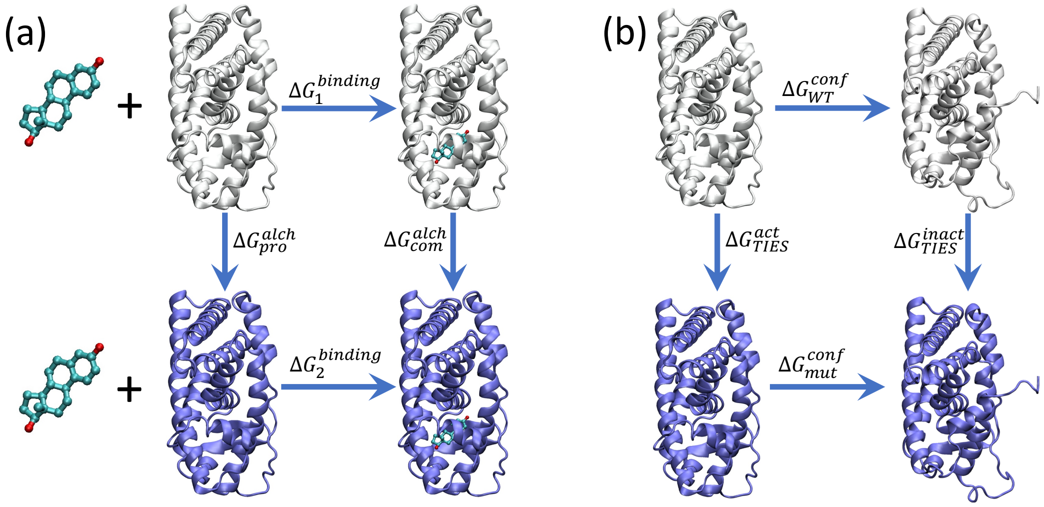

Figure S1. Thermodynamic cycles used in the study to calculate binding free energies or binding free energy differences (a), and conformational free energy changes (b). (a) The horizontal lines are the binding processes of a compound to ER with specific sequences such as the wild-type and a mutation (indicated in silver and blue colours, respectively); The vertical lines are alchemical processes during which the protein is transferred from one sequence (wild-type, silver) to another (mutant, blue). For the calculation of the conformational free energy changes (b), the vertical lines have the same meaning as these in (a), but at two different conformational states (active and inactive); the horizontal lines are conformational changes from active to inactive states.

Ensemble approaches. Ensemble-based methods are the central focus of our attention since these provide the correct statistical-mechanical way in which to calculate macroscopic quantities such as free energies from microscopic dynamics[1,4]. Extensive studies we have performed in recent years have shown that ensemble approaches are able to deliver predictions accurately and reproducibly[2,5-11]. The predictions are also made on time scales that are sufficiently rapid to be used in a clinical decision-making context. This is essential for such techniques to become a standard technique applicable in diverse applications, including pharmaceutical and clinical contexts. Here we use the ensemble-based ESMACS and TIES approaches for the binding free energy calculations of drugs to wild-type and mutant estrogen receptors.

ESMACS protocol. In ESMACS, the free energy is evaluated approximately based on the extended MMPBSA (Molecular Mechanics Poisson-Boltzmann Surface Area) method, including configurational entropy, and the free energy of association. Default dielectric constants are used: 1 for interior (dielectric in the solute) and 80 for exterior (dielectric in the bulk). Our previous studies have shown that the sampled conformations are extensively enhanced in the ensemble simulations than these in single simulations[1-3,5,6,12,13]. According to the protocol we have developed, an ensemble consisting of 25 replica simulations is used for each complex in order to provide uncertainty quantification at the level of about 1 kcal/mol. The number of replicas and the simulation length of each replica are carefully determined[3,12] so that an optimal trade-off between precision and computational cost is achieved. The configurational entropy calculations can be performed for the entire complex/protein/ligand systems. Our calculations show that the configurational entropy component does not contribute to the quality of the free energy prediction. This is not surprising as different conclusions – improving, worsening, or having no effect – have been drawn for diverse protein−ligand complexes[8]. The binding free energies reported here therefore do not include the configurational entropies.

Table S1. Summary of the 1-, 2- and 3-trajectory methods, in which trajectories of individual components are extracted from ensemble simulations of the complex (C) or separate simulations of the receptor (R) and ligands (L).

Protocol	Complex	Receptor	Ligand
1-traj	C	C	C
2-traj	C	R	C
	C	C	L
3-traj	C	R	L

ESMACS can be performed using ensembles of 1-, 2- and/or 3-trajectory methods (Table S1). In the 1-trajectory approach, the trajectories for all of the three components – the complexes, the receptors and the ligands – are extracted from the simulations of the complexes. In the 2-trajectory, individual simulations are performed for two of the components, either complexes and receptors, or complexes and ligands; the trajectories are extracted from the corresponding simulations. For the component for which simulations are not performed (receptor or ligand), the trajectories are extracted from the complex simulations. In the 3-trajectory methods, simulations are performed for each of the components, and trajectories are extracted from their individual simulations. It is highly informative to compare findings based on the 1-, 2- and 3-trajectory versions of ESMACS[3,7,8]. For ligand-protein binding that approximates well the

original lock-and-key hypothesis, 1-trajectory ESMACS will work well; for situations involving "induced fits" and other steric considerations, 2- and/or 3-trajectory ESMACS is required and is capable of providing many detailed insights into the binding energetics. In the current study, the 1-trajectory method works well, indicating that the ligands bind in a lock-and-key fashion to the estrogen receptor. It should be noted that the activation of estrogen receptor involves relatively large conformational changes, especially for H12 of which the orientation changes significantly. The inference of a lock-and-key binding motif is based on an assumption of conformational-selection binding.

TIES protocol. The binding free energy difference, $\Delta\Delta G_{binding}$, of two ligands to the same protein, or a ligand to wild-type and variant proteins, can be computed using an alchemical transformation for the mutated entity in aqueous solution and within the ligand-protein complex. In an alchemical transformation, some atoms gradually vanish from the initial state, while some slowly appear towards the final state. In the simulations, a hybrid molecule is constructed which consists of both the initial and final states. A coupling parameter λ ($0 \leq \lambda \leq 1$) is introduced to describe an intermediate state λ, where λ=0 and λ=1 correspond to the initial and final thermodynamic states. In order to avoid "end-point catastrophes"[14], a soft-core potential is used for pairwise van der Waals interactions involving the perturbed atoms.

We have extended the TIES methodology to study relative binding affinities caused by the protein mutations when bound to a ligand, a variant which we call TIES-PM[5]. TIES-PM is conceptually identical to TIES, except that the alchemically transforming region includes the mutating protein residue. Corrections for electrostatic finite-size effects have been added to the TIES-PM approach[6], extending the domain of applicability of the method to cases where a change of net charge between the pair of variants occurs along the alchemical path used in thermodynamic integration.

The convergence analyses of our previous studies show that 5 replicas and 4 ns simulation per replica at each λ window are sufficient to generate precise results[2,15], and are hence used in the current study. Thirteen λ windows, consisting of the two endpoints representing the two physical states (wild-type and mutant ERs) and 11 intermediate alchemical states, were simulated for the alchemical process of the mutations. The intermediate windows were mixtures of the two physical states (Eq. S3). Five replicas were used for each λ window, from which the energy deviations and the statistical errors were calculated.

Simulation setup. Ensembles of 25 replicas for ESMACS, and 5 replicas for each λ window in TIES, were conducted using the package NAMD 2.12[16]. Amber and AmberTools[17] were used to set up and analyse of the simulations. Protonation states of all titratable residues were assigned at neutral pH using the reduce algorithm[18], and checked virtually for the residues near the binding site. The non-bonded cutoff was 12 Å, and the Particle Mesh Ewald (PME) method was used to treat the long-range Coulomb interactions. All simulations were performed using the protocol incorporated in the binding affinity calculator (BAC)[19]. Each replica was first minimized with all heavy protein atoms restrained at their initial positions. The initial velocities were then generated independently from a Maxwell–Boltzmann distribution at 50 K, and the systems were heated up to 300 K. Once the system reached the correct temperature, an isothermal–isobaric ensemble (NPT) was maintained with a temperature of 300 K and a pressure of 1 bar. A series of equilibration runs, totaling 2 ns, were conducted, while the restraints on heavy atoms were gradually reduced. Finally, 4 ns production simulations were run for each replica for all ESMACS and TIES simulations. A 2 fs time step was used for all MD simulations. Trajectories were recorded every 10 ps in the production runs.

For ESMACS, the trajectories were further analysed by MMPBSA.py.MPI to extract the energetic information for each snapshot. A script was then run to aggregate these results from

the ensemble of simulations and values of ΔG_{ESMACS} computed along with bootstrap statistics. In TIES, energy derivatives were recorded during MD simulations, which were integrated using a trapezoidal rule to obtain the alchemical free energy ΔG_{alch}. Some of the drugs contain polar moieties which form hydrogen bonds with protein residues as well as water molecules in the binding site. While the water molecules were explicitly included in the simulations, their contributions to the binding free energies were taken into account explicitly in TIES-PM calculations, and implicitly in ESMACS calculations.

Hydrogen-bond analyses were performed for the trajectories of the wild-type and mutant proteins at both active and inactive states. A hydrogen bond was considered to be formed when the distance between a hydrogen-bond acceptor and a hydrogen-bond donor was less than a defined distance cutoff and the acceptor−hydrogen−donor angle was greater than an angle cutoff. The module cpptraj of AmberTools[17] was used, with the default distance cutoff of 3.0 Å and the angle cutoff of 135°. The frequency of occurrence was reported as the percentage of snapshots in which a specified hydrogen bond was observed.

Standard errors and convergence. Ensemble averaging[1] was used to compute means and bounded standard errors of the calculated macroscopic free energies. Using the data from ensemble simulations, the macroscopic binding affinities were estimated with well-defined mean and bounded standard error. To do this, we used bootstrap resampling to resample (with replacement) the data from our sample of 25 values and calculate the corresponding mean. A very large number of bootstrap samples (100,000 in our studies) was generated, drawing with replacement from the population of the original data set. "Resample with replacement" allowed some data in the original set to appear more than once, while others did not appear at all. The standard deviation of the means provided an estimate of the standard error in the calculated binding affinity (ΔG). The variances of ΔΔG values from ESMACS in Table 2 were the sum of the variances of two independent ΔG values.

The error analyses for TIES were done in a similar way to that in ESMACS. The standard errors, σ_λ, were evaluated for each λ window using the bootstrap resampling as mentioned above. The error associated with each alchemical free energy calculation, σ_{alch}, was computed using:

$$\sigma_{alch}^2 = \sum_{\lambda} \sigma_\lambda^2 (\Delta\lambda)^2$$

where σ_λ^2 was the variance associated with the relevant λ-window in the aqueous or bound calculation. The overall error, σ, for the free energy difference (ΔΔG) was then computed using:

$$\sigma^2 = \sigma_{alch\_aqueous}^2 + \sigma_{alch\_bound}^2$$

Mutations Identified from Sequencing Analysis

Variant-calling computational analysis was performed on the sequenced data obtained from 50 breast cancer samples and missense variants were identified. These missense single nucleotide polymorphism (SNP) variants in the sequenced data were called by SeqScape Software 3 (applied biosystems). A mutation report was generated for each patient. Chromatogram analysis was performed on the sequenced data to detect artifacts/mis-called-nucleotides and aberrations. In this manner, a list of SNPs was generated consisting of the patient number, mutation and its novelty or known status from variant databases such as dbSNP[20]. The variants in SNP list were identified in the cDNA of the ESR1 reference manually. From our

sequencing study 22 nonsynonymous mutations were identified (see Table S2). Modelling and analysis of nsSNPs was performed in StSNP[21].

Among the identified 22 mutations, 14 mutations were noted to be novel with no annotations available in nucleotide variants repositories and 9 mutations were found to be known with their respective annotations accessible in variant databases such as dbSNP. Frequencies of detected variants in the studied 50 breast cancer samples were computed to understand the occurrence and cluster pattern of variants across the analysed patient cohort (see Table S3). The study of variants' occurrence and clustering patterns could be used for further statistical analysis to detect unique and prevalent variants among diverse ethnic population thus aiding the goal of precision medicine in pharmacology. Additionally, chromatogram quality of the identified mutations signifying the possible real or possible artefact status of respective mutations is also represented in Table S3.

As can be seen in Table S3, the mutations with the highest frequency among these patients are annotated ones. Looking at the sequencing accuracy and chromatogram quality, we observe that the highest frequency mutations at positions 10, 243 and 325 are "clear", conferring significant accuracy of the bases called by the sequencer. However, other mutations in this table exhibit variations in the significance of their being true mutations or mis-called mutations/artefacts.

Among the mutations studied using modelling analysis we have the following:

a) the mutation at position 387 was found to be a possible artefact because of unclear chromatogram;
b) the mutation at position 384 was identified as a possible real mutation due to clear chromatogram;
c) the mutation at position 485 was observed with a clear chromatogram and possibly real;
d) the mutations at positions 529 and 548 were identified as possible artefacts due to unclear chromatograms.

Table S2. Identified mutations in the ESR1 gene.

No	Mutation	Freq	Database	rs# for mutation in database	Known/Novel Status	Chromosome position
1	S/S 10	20	dbSNP	rs2077647	known	6:151807942
2	P/H 55	1	-	-	novel	6:151808076
3	A/A 87	4	dbSNP	rs746432	known	6:151808173
4	P/S 147	1	-	-	novel	6:151808351
5	R/R 243	30	dbSNP	rs4986934	known	6:151880740
6	S/F 282	3	-	-	novel	6:151944257
7	A/A 318	1	dbSNP	rs1401125809	known	6:151944366
8	P/P 325	30	dbSNP	rs1801132	known	6:151944387
9	L/V 384	1	-	-	novel	6:152011709
10	T/A 431	1	dbSNP	rs1204611622	known	6:152061046
11	T/I 485	1	-	-	novel	6:152094469
12	T/T 594	7	dbSNP	rs2228480	known	6:152098960
13	H/Y 16	1	-	-	novel	6:151807958
14	T/S 140	1	-	-	novel	6:151808330
15	R/S 162	1	-	-	novel	6:151842630
16	D/A 170	1	-	-	novel	6:151842653
17	K/K 210	1	COSMIC	COSM4992439	known	6:151842774
18	C/F 221	1	-	-	novel	6:151880673
19	R/P 269	1	-	-	novel	6:151944218
20	L/R 387	1	-	-	novel	6:152011719
21	K/N 529	1	-	-	novel	6:152098765
22	R/P 548	1	-	-	novel	6:152098821

Table S3. Frequency, and reall or artefact status of variants identified in 50 breast cancer patient samples. (K = Known; N = Novel; Chromatogram clear = possible functional mutation; Chromatogram unclear = possible artefact).

Mutation Position	Known/ Novel Status	Frequency	Patient Number	Chromatogram Quality	Possible-real/ Possible-artefact
16(H/Y)	N	1	4	unclear	artefact
55(P/H)	N	1	15	clear	real
87(A/A)	K	4	34,36,38,45	clear	real
140(T/S)	N	1	48	unclear	artefact
147(P/S)	N	1	2	clear	real
162(R/S)	N	1	20	unclear	artefact
170(D/A)	N	1	20	unclear	artefact
210(K/K)	K	1	15	unclear	artefact
221(C/F)	N	1	26	unclear	artefact
269(R/P)	N	1	44	unclear	artefact
282(S/F)	N	3	15,38,49	clear	real
318(A/A)	K	1	5	clear	real
384(L/V)	N	1	39	clear	real
387(L/R)	N	1	33	unclear	artefact
431(T/A)	K	1	27	unclear	artefact
485(T/I)	N	1	10	clear	real
529(K/N)	N	1	24	unclear	artefact
548(R/P)	N	1	22	unclear	artefact
594(T/T)	K	7	3,17,36,40,46,47,49	clear	real
243(R/R)	K	30	5,10,14,15,16,17,19,21,26,27,28,31,33,34,35,36,37,38,39,40,41,42,43,44,45,46,47,48,49,50	clear	real
10(S/S)	K	20	10,13,15,16,26,30,31,36,37,38,39,41,42,43,44,45,46,47,48,49	clear	real
325(P/P)	K	30	3,5,8,9,12,15,17,18,21,22,23,25,26,29,31,33,34,35,36,37,38,39,40,42,43,45,47,48,49,50	clear	real

References

1 Coveney, P. V. & Wan, S. On the calculation of equilibrium thermodynamic properties from molecular dynamics. *Phys Chem Chem Phys* **18**, 30236-30240, doi:10.1039/c6cp02349e (2016).

2 Bhati, A. P., Wan, S., Wright, D. W. & Coveney, P. V. Rapid, Accurate, Precise, and Reliable Relative Free Energy Prediction Using Ensemble Based Thermodynamic Integration. *J Chem Theory Comput* **13**, 210-222, doi:10.1021/acs.jctc.6b00979 (2017).

3 Wan, S., Knapp, B., Wright, D. W., Deane, C. M. & Coveney, P. V. Rapid, precise, and reproducible prediction of peptide-MHC binding affinities from molecular dynamics that correlate well with experiment. *J Chem Theory Comput* **11**, 3346-3356, doi:10.1021/acs.jctc.5b00179 (2015).
4 Knapp, B., Ospina, L. & Deane, C. M. Avoiding False Positive Conclusions in Molecular Simulation: The Importance of Replicas. *J Chem Theory Comput* **14**, 6127-6138, doi:10.1021/acs.jctc.8b00391 (2018).
5 Bhati, A. P., Wan, S. & Coveney, P. V. Ensemble-Based Replica Exchange Alchemical Free Energy Methods: The Effect of Protein Mutations on Inhibitor Binding. *J Chem Theory Comput* **15**, 1265-1277, doi:10.1021/acs.jctc.8b01118 (2019).
6 Bhati, A. P., Wan, S., Hu, Y., Sherborne, B. & Coveney, P. V. Uncertainty Quantification in Alchemical Free Energy Methods. *J Chem Theory Comput* **14**, 2867-2880, doi:10.1021/acs.jctc.7b01143 (2018).
7 Wan, S. *et al.* Evaluation and Characterization of Trk Kinase Inhibitors for the Treatment of Pain: Reliable Binding Affinity Predictions from Theory and Computation. *J Chem Inf Model* **57**, 897-909, doi:10.1021/acs.jcim.6b00780 (2017).
8 Wan, S. *et al.* Rapid and Reliable Binding Affinity Prediction of Bromodomain Inhibitors: A Computational Study. *J Chem Theory Comput* **13**, 784-795, doi:10.1021/acs.jctc.6b00794 (2017).
9 Wan, S., Tresadern, G., Pérez-Benito, L., Vlijmen, H. & Coveney, P. V. Accuracy and Precision of Alchemical Relative Free Energy Predictions with and without Replica-Exchange. *Adv Theory Simul* **3**, 1900195, doi:10.1002/adts.201900195 (2019).
10 Wright, D. W. *et al.* Application of the ESMACS Binding Free Energy Protocol to a Multi-Binding Site Lactate Dehydogenase A Ligand Dataset. *Adv Theory Simul* **3**, 1900194, doi:10.1002/adts.201900194 (2019).
11 Wright, D. W. *et al.* Application of ESMACS binding free energy protocols to diverse datasets: Bromodomain-containing protein 4. *Sci Rep* **9**, 6017, doi:10.1038/s41598-019-41758-1 (2019).
12 Wright, D. W., Hall, B. A., Kenway, O. A., Jha, S. & Coveney, P. V. Computing Clinically Relevant Binding Free Energies of HIV-1 Protease Inhibitors. *J Chem Theory Comput* **10**, 1228-1241, doi:10.1021/ct4007037 (2014).
13 Wright, D. W. *et al.* Application of the ESMACS Binding Free Energy Protocol to a Multi-Binding Site Lactate Dehydogenase A Ligand Dataset. *Adv Theory Simul* **n/a**, 1900194, doi:10.1002/adts.201900194 (2019).
14 Beveridge, D. L. & Dicapua, F. M. Free-energy via molecular simulation - applications to chemical and biomolecular systems. *Annu Rev Biophys Bio* **18**, 431-492, doi:DOI 10.1146/annurev.bb.18.060189.002243 (1989).
15 Wan, S., Tresadern, G., Pérez-Benito, L., van Vlijmen, H. & Coveney, P. V. Accuracy and Precision of Alchemical Relative Free Energy Predictions with and without Replica-Exchange. *Adv Theory Simul* **n/a**, 1900195, doi:10.1002/adts.201900195 (2019).
16 Phillips, J. C. *et al.* Scalable molecular dynamics with NAMD. *J Comput Chem* **26**, 1781-1802, doi:10.1002/jcc.20289 (2005).
17 Case, D. A. *et al.* The Amber biomolecular simulation programs. *J Comput Chem* **26**, 1668-1688, doi:10.1002/jcc.20290 (2005).
18 Word, J. M., Lovell, S. C., Richardson, J. S. & Richardson, D. C. Asparagine and glutamine: using hydrogen atom contacts in the choice of side-chain amide orientation. *J Mol Biol* **285**, 1735-1747, doi:10.1006/jmbi.1998.2401 (1999).
19 Sadiq, S. K. *et al.* Automated molecular simulation based binding affinity calculator for ligand-bound HIV-1 proteases. *J Chem Inf Model* **48**, 1909-1919, doi:10.1021/ci8000937 (2008).
20 Sherry, S. T. *et al.* dbSNP: the NCBI database of genetic variation. *Nucleic Acids Res* **29**, 308-311, doi:10.1093/nar/29.1.308 (2001).
21 Uzun, A., Leslin, C. M., Abyzov, A. & Ilyin, V. Structure SNP (StSNP): a web server for mapping and modeling nsSNPs on protein structures with linkage to metabolic pathways. *Nucleic Acids Res* **35**, W384-392, doi:10.1093/nar/gkm232 (2007).
